# Cracking the Hard Seed: Molecular Mechanisms and Multi-Omics Insights into Seed Dormancy and Germination in the Genus *Astragalus*

**DOI:** 10.3390/ijms27146312

**Published:** 2026-07-15

**Authors:** Waqar Afzal Malik, Maria Afzal, Salsabeel Yousuf, Li Ma, Xinchun Tian, Lei Wang, Yunling Zhang, Hongling Wang

**Affiliations:** 1 Key Laboratory of Ecological Safety and Sustainable Development in Arid Lands, Xinjiang Institute of Ecology and Geography, Chinese Academy of Sciences, Urumqi 830011, China; waqarviqi244@gmail.com (W.A.M.); mariaafzal244@gmail.com (M.A.); egiwang@ms.xjb.ac.cn (L.W.); 2 Agricultural Genomics Institute at Shenzhen, Chinese Academy of Agricultural Sciences, Shenzhen 518120, China; sabeelstar@gmail.com; 3 General Grassland Station of Xinjiang, Urumqi 830049, China; mary563829@163.com (L.M.); 15099176765@189.cn (X.T.); 4 Research Center for Ecology and Environment of Central Asia, Xinjiang Institute of Ecology and Geography, Chinese Academy of Sciences, Urumqi 830011, China

**Keywords:** *Astragalus*, seed dormancy, physical dormancy, hardseededness, germination, seed coat, ABA, gibberellin, multi-omics, transcriptomics, flavonoids, astragalosides

## Abstract

The genus *Astragalus* comprises one of the largest and most ecologically diverse groups of flowering plants and includes species of major medicinal, forage, and restoration value, particularly *Astragalus membranaceus* and allied taxa. However, effective germplasm utilization and large-scale cultivation remain constrained by strong seed dormancy, most commonly expressed as physical dormancy imposed by a water-impermeable seed coat. In some species, this coat-imposed barrier is further complicated by an additional physiological component, resulting in combinational dormancy. This review synthesizes current knowledge on the structural, molecular, and multi-omics basis of seed dormancy and germination in *Astragalus*, with emphasis on the dormancy-to-germination transition. We first examine the anatomical basis of hardseededness, including the palisade layer, light line, and associated seed-coat barrier domains, and discuss the likely contributions of phenylpropanoid, lignin, suberin, and cutin biosynthesis to the establishment of physical dormancy. We then summarize current understanding of hormone crosstalk, highlighting the central roles of abscisic acid and gibberellins together with ethylene, brassinosteroids, reactive oxygen species, and nitric oxide in regulating post-dormancy germination. Recent transcriptomic, metabolomic, and emerging integrative omics studies reveal that *Astragalus* germination involves not only reserve mobilization and energy activation but also early induction of characteristic secondary-metabolite pathways, particularly flavonoids and isoflavonoids, which distinguishes medicinal *Astragalus* seeds from many conventional crop seeds. Finally, we discuss current bottlenecks, including the limited availability of robust functional-genomics tools, and outline future directions involving CRISPR-based validation, epigenetic regulation, and marker-assisted breeding for improved germination uniformity and reduced hardseededness. This review provides a mechanistic framework for unlocking *Astragalus* seed dormancy and accelerating the conservation, breeding, and medicinal utilization of this important genus.

## 1. Introduction

The genus *Astragalus* L. is one of the most species-rich lineages of angiosperms and the largest genus in Fabaceae by many taxonomic treatments, comprising nearly 3000 species distributed largely across temperate, arid, and semi-arid regions of the Northern Hemisphere [1,2]. This exceptional diversity is matched by substantial ecological breadth. *Astragalus* species occur in steppes, deserts, alpine systems, and dry rangelands, where they often represent conspicuous components of local vegetation and contribute to the persistence of plant communities through durable soil seed banks and stress-adapted reproductive strategies [2]. At the same time, the genus has remarkable economic and ethnobotanical importance. Several species are valued as forage or restoration plants, while others are central to traditional medicine. Among them, *A. membranaceus* and its allied taxon *A. mongholicus* are especially prominent because their roots, widely recognized as Astragali Radix or Huangqi, have long been used in Asian medical systems and are now the focus of extensive phytochemical and pharmacological research [1,3]. Their medicinal value is linked to a rich repertoire of bioactive metabolites, notably polysaccharides, flavonoids, and triterpenoid saponins, which have been associated with immunomodulatory, antioxidant, anti-inflammatory, and other therapeutic activities [1,3].

Despite this ecological and medicinal importance, the propagation biology of *Astragalus* remains a major practical challenge, and seed dormancy lies at the center of that challenge. In wild habitats, dormancy is an adaptive trait that prevents immediate germination under transiently favorable but ultimately unsafe conditions, thereby spreading recruitment through time and buffering populations against environmental unpredictability [2,4]. For many *Astragalus* species that inhabit climatically harsh landscapes, such delayed germination is likely to be a critical component of persistence. However, the same trait becomes a bottleneck in agriculture, medicinal plant cultivation, ecological restoration, ex situ conservation, and germplasm regeneration, where rapid, synchronized, and predictable seedling emergence is often required [5,6]. Hardseededness can sharply reduce germination uniformity, complicate nursery establishment, and obscure the distinction between truly nonviable seed and viable but dormant seed. Thus, in *Astragalus*, dormancy is best understood as both an evolutionary solution and an applied constraint: it secures survival in nature while limiting efficient utilization by breeders, conservationists, and producers [2,5,6].

Within the Baskin and Baskin dormancy framework, seeds may express several dormancy classes, including physiological dormancy (PD), physical dormancy (PY), and combinational dormancy (PY + PD) [4]. Available evidence indicates that *Astragalus* is, as a genus, predominantly characterized by physical dormancy. The most consistent pattern reported across species is the presence of a water-impermeable seed coat, with dormancy effectively broken by mechanical or acid scarification, indicating that the primary barrier to germination is imposed by seed-coat structure rather than embryo incapacity alone [5,6,7]. A meta-analysis focused specifically on *Astragalus* concluded that most species likely possess PY and that scarification is generally the most effective treatment for dormancy release [5]. Individual species studies support this interpretation; for example, *A. arpilobus* exhibits a classic hardseeded phenotype in which most seeds remain impermeable until scarified [7]. Nonetheless, PY does not explain every case. In some taxa, germination responses suggest that a physiological block may remain after the water barrier is removed. This pattern has been demonstrated in *A. filipes*, where dormancy behavior was interpreted as combinational dormancy requiring both seed-coat disruption and physiological after-treatment [8]. Accordingly, the most defensible genus-level synthesis is that *Astragalus* seed dormancy is predominantly physical, with confirmed but taxonomically uneven occurrences of PY + PD [4,5,8].

This dormancy complexity creates an important conceptual gap. While ecological and applied studies have identified many effective dormancy-breaking treatments, the molecular basis of the transition from a hard, quiescent seed to an actively germinating one remains incompletely resolved in *Astragalus*. Key questions are still open: how seed-coat architecture is established and weakened, how hormonal circuits such as abscisic acid and gibberellin signaling are integrated after permeability is gained, how reactive oxygen balance and reserve mobilization are coordinated, and which transcriptional and metabolic switches determine whether an imbibed seed proceeds to radicle protrusion or remains arrested. These issues are particularly important for *Astragalus*, because dormancy release is not merely a physiological event but a gatekeeping step for medicinal crop establishment, germplasm multiplication, and trait improvement.

**Figure 1:**
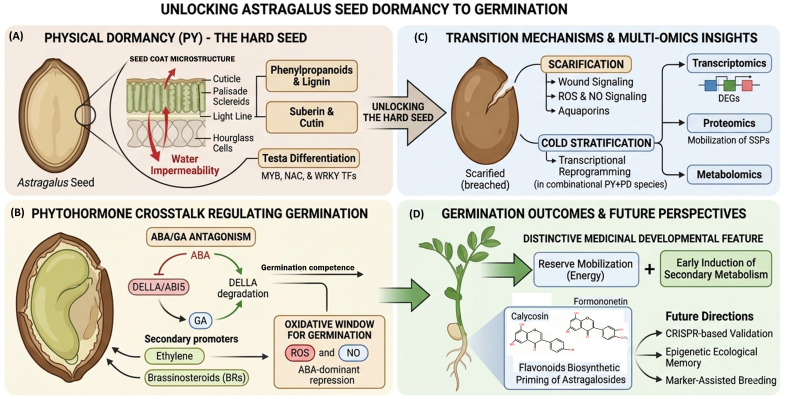
Integrated model of seed dormancy release and germination in *Astragalus*. (**A**) Seed coat-imposed physical dormancy. The hard seed coat comprises an outer cuticle, a palisade layer (macrosclereids) with a characteristic light line in its outer region, underlying hourglass cells, and inner parenchyma, conferring water impermeability. This structure is regulated by MYB, NAC, and WRKY transcription factors. (**B**) Dormancy-breaking treatments and water entry. Antagonism between ABA and GA involves *DELLA/ABI5* repression and DELLA degradation, establishing an oxidative window (ROS/NO) that overcomes ABA-dominant repression. (**C**) Multi-omics transition from dormancy to germination. Transcriptomics, proteomics, and metabolomics reveal reprogramming of hormone signaling, reserve mobilization, and secondary metabolism. (**D**) Germination outcomes, medicinal metabolism, and future applications. The transition involves energy activation coupled with early induction of flavonoids/isoflavonoids. Future perspectives point to CRISPR-based validation, epigenetic ecological memory, and marker-assisted breeding.

Full-length transcriptome reconstruction in *A. membranaceus* has revealed extensive transcript diversity associated with bioactive compound biosynthesis [9]. More recently, a reference-grade genome for *A. mongholicus* and a chromosome-scale genome assembly for *A. membranaceus* have created a stronger foundation for gene discovery, comparative analysis, and pathway-level interpretation [10,11]. In parallel, integrated transcriptome-metabolome analysis has already begun to illuminate dormancy-related developmental transitions in *Astragalus*, albeit so far mainly in underground bud dormancy rather than seed dormancy sensu stricto [12]. These advances make it possible to revisit the long-standing problem of hardseededness from a multi-omics perspective. In this review, we therefore examine seed dormancy and germination in *Astragalus* through four linked lenses: the ecological and structural basis of dormancy, the molecular mechanisms that govern dormancy release and germination, emerging omics evidence and candidate regulatory networks, and the translational implications for germplasm utilization, domestication, and medicinal crop improvement. By doing so, we aim to move the discussion beyond treatment-based germination protocols toward a mechanistic understanding of the dormancy-to-germination transition in this ecologically important and pharmaceutically valuable genus [10,11,12]. The overall conceptual structure of this review, linking seed coat-imposed dormancy, dormancy-breaking cues, hormone-redox signaling, multi-omics reprogramming, medicinal-metabolite activation, and translational applications, is summarized in Figure 1.

## 2. Structural and Molecular Basis of Seed Coat-Imposed Dormancy (PY)

In *Astragalus*, as in many hard-seeded legumes, physical dormancy (PY) arises from a specialized seed coat that restricts water entry until environmental or artificial cues disrupt that barrier [13,14]. Although the structural basis of hardseededness in *Astragalus* is well supported by anatomical and dormancy-breaking studies, the underlying gene networks remain far less resolved at the genus level. Accordingly, current understanding is best built by combining direct *Astragalus* evidence with mechanistic insight from closely related legume systems and from seed coat studies in model plants [6,13,14]. The main structural and molecular components proposed to underlie seed coat-imposed dormancy in *Astragalus* are illustrated in Figure 1A.

### 2.1. Microstructure of the Astragalus Seed Coat: The Water-Impermeable Palisade Layer and Light Line

The structural hallmark of legume PY is a dense outer testa dominated by elongated palisade macrosclereids, typically covered by a cuticular layer and underlain by hourglass cells and inner parenchymatous tissues [6,14]. Across legumes, this palisade zone is widely regarded as the principal anatomical barrier to imbibition, especially when reinforced by hydrophobic phenolics, suberin-associated materials, waxes, and tightly organized cell walls [6]. A characteristic “light line” traversing the palisade layer has also long been associated with impermeability in hard seeds, and in classic legume studies it has been treated as part of the major diffusion barrier to water [15]. In *Astragalus*, available evidence strongly supports the same overall architectural logic, even though detailed ultrastructural descriptions of the light line itself remain much scarcer than the broader documentation of palisade-based impermeability [6,13]. These anatomical features are represented schematically in Figure 1A, where the palisade layer, light line, and associated seed coat tissues are positioned as the primary physical barrier to water entry.

Among *Astragalus* species for which detailed anatomical and physiological studies are available, *A. adsurgens* provides the most direct mechanistic evidence regarding the onset of physical dormancy. Jaganathan et al. showed that freshly matured seeds were initially permeable, but became impermeable after post-harvest drying once seed moisture content dropped below a critical threshold, demonstrating that PY in this species is not simply a static trait of seed thickness but a dehydration-dependent sealing state [13]. While the genus encompasses nearly 3000 species with considerable ecological and anatomical diversity, the broader *Astragalus* literature consistently supports physical dormancy as the predominant dormancy class, as confirmed by a genus-wide meta-analysis [5]. However, the species-specific variation in the precise structural and molecular mechanisms of dormancy establishment is likely. Thus, *A. adsurgens* is best viewed as an informative case study that illustrates the dehydration-dependent sealing phenomenon, rather than as a representative model for the entire genus. Further, scanning electron microscopy identified the principal papilionoid seed-coat landmarks, including the hilum, micropyle, lens, and extra-hilar region, and showed that sulfuric acid treatment progressively opened discrete surface regions through which water could subsequently enter [13]. Importantly, water entry in *A. adsurgens* appeared to be associated mainly with the hilar and extra-hilar regions rather than a single universal water-gap site, indicating that *Astragalus* may use structurally heterogeneous weak points for dormancy break [13]. This is consistent with the broader legume literature, where permeability can be regulated by the lens, strophiole, hilum, micropyle, or local fissures depending on species [6,13]. The proposed transition from an impermeable seed coat to localized water entry through structurally vulnerable regions is summarized in Figure 1B.

Taken together, the available evidence supports a working structural model for *Astragalus* PY in which the palisade layer functions as the principal water-exclusion barrier, while specialized regions such as the hilum, lens, or adjacent extra-hilar tissues act as conditional entry points that can be activated by drying, temperature fluctuation, or scarification [6]. In practical terms, this explains why mechanical scarification, acid scarification, or other testa-disruptive treatments are often highly effective in overcoming dormancy in *Astragalus* seeds. It also implies that structural dormancy release is likely governed not only by gross seed-coat thickness, but by the chemistry and integrity of localized barrier domains.

### 2.2. Molecular Regulation of the Phenylpropanoid Pathway and Lignin Biosynthesis

At the molecular level, one of the most plausible determinants of *Astragalus* hardseededness is the phenylpropanoid–lignin network that strengthens and hydrophobizes the outer testa. However, direct developmental expression datasets for *Astragalus* seed coats, especially for canonical lignin-pathway genes such as PAL (phenylalanine ammonia-lyase), C4H (cinnamate 4-hydroxylase), 4CL (4-coumarate:CoA ligase), and CAD (cinnamyl alcohol dehydrogenase), are still limited [15]. Current inference therefore depends heavily on other hard-seeded systems. In vegetable soybean, genome-wide transcriptome analysis linked seed hardness to enhanced expression of multiple phenylpropanoid and lignin biosynthetic genes, including 4CL, CAD, HCT, and CCoAOMT, during critical stages of seed development [16]. Likewise, comparative analyses of Brassica napus seed coats with contrasting lignin contents showed stronger expression of PAL- and C4H-family genes in high-lignin seed coats, reinforcing the idea that differential flux through the upstream phenylpropanoid pathway contributes directly to testa hardening [17].

These observations are highly relevant to *Astragalus*. In a hard seed, lignification is not merely a mechanical reinforcement process. It also affects apoplastic porosity, wall densification, and the interaction between aromatic polymers and lipidic barriers. From this perspective, PAL, C4H, 4CL, and CAD can be viewed as core candidate regulators of the transition from a permeable developing testa to a mature water-resistant seed coat. PAL and C4H determine the entry and early commitment of carbon into the phenylpropanoid pathway; 4CL activates hydroxycinnamate intermediates for downstream branch metabolism; and CAD contributes to the final reduction steps required for monolignol production. A developmentally elevated flux through this module would be expected to increase deposition of lignified or phenolic wall material in palisade cells and related seed-coat tissues, thereby promoting impermeability [16,17].

A further point of importance is that seed impermeability in legumes appears to depend on seed-coat composition rather than on lignin alone. Studies comparing soybean seeds with contrasting permeability have shown that water-impermeable seed coats differ in chemical composition and barrier integrity, supporting the broader view that the phenylpropanoid pathway acts together with other structural and lipidic processes [6,18]. Thus, in *Astragalus*, the most reasonable mechanistic hypothesis is that phenylpropanoid activation contributes to a composite barrier system in which lignified palisade walls, phenolic impregnation, and lipid polyester deposition jointly determine whether the testa remains hard or becomes permeable. This hypothesis now needs direct testing through seed-coat-specific transcriptomics, histochemical lignin mapping, and targeted quantification of phenylpropanoid intermediates across dormancy acquisition and release stages in *Astragalus* species.

### 2.3. Lipid Metabolism: Suberin and Cutin Deposition Mediated by Cytochrome P450s and Lipid Transfer Proteins

The seed coat barrier in physically dormant legumes also depends strongly on lipid metabolism, particularly the biosynthesis and assembly of cutin- and suberin-like polymers that reduce wettability and diffusion [6,14]. Recent work in *Medicago truncatula* has been especially informative. Chai et al. demonstrated that the class II KNOX transcription factor *KNOX4* is essential for seed physical dormancy because it governs proper seed-coat cuticle development; loss of *KNOX4* produced defective palisade cuticles and seeds that readily absorbed water [19]. In a later study, the same group showed that *KCS12*, a seed-coat-specific β-ketoacyl-CoA synthase required for very-long-chain fatty acid synthesis, is critical for preserving physical dormancy, again linking testa impermeability to the biosynthesis of specialized lipids [20]. Even more recently, *Anthocyanidin reductase* (*Anr*) was found to promote physical dormancy in *M. truncatula*, revealing unexpected crosstalk between flavonoid metabolism and seed-coat lipid composition [21]. Together, these studies make it clear that PY is not simply a lignin problem. It is also a lipid-barrier problem.

Mechanistically, this lipidic barrier likely depends on a conserved biosynthetic toolkit that is highly relevant to *Astragalus*. In *Arabidopsis*, *GPAT5* is required for seed-coat and root suberin polyester formation, and gpat5 mutants exhibit sharply increased seed permeability [22]. *CYP86B1*, a cytochrome P450 fatty acid ω-hydroxylase, is required for the formation of very-long-chain ω-hydroxyacids and α,ω-dicarboxylic acids in seed suberin polyester, defining an essential enzymatic step in hydrophobic barrier assembly [23]. In parallel, the GPI-anchored lipid transfer protein *LTPG15* has been shown to affect seed-coat permeability by participating in the export of suberin-related monomers [24]. These findings strongly suggest that orthologous *CYP86*, GPAT, KCS, and lipid transfer protein modules should be considered prime candidates in *Astragalus* hardseed biology, even though they have not yet been functionally validated there.

In *Astragalus*, therefore, lipid metabolism should be viewed as a central rather than auxiliary component of PY. The mature water barrier probably emerges from the coordinated deposition of cuticular and suberin-associated aliphatics across the outer testa, especially around palisade cells and water-gap domains. Once this lipid matrix is disrupted, either chemically by acid scarification or developmentally by weakening of its synthesis or export, water entry becomes possible. A key future priority is to determine whether hard-seeded and less hard-seeded *Astragalus* taxa differ in the abundance, chain length, oxidation state, and localization of seed-coat aliphatics, and whether such differences track the developmental expression of *CYP86A*/B, *GPAT5*-like, *KCS12*-like, and LTP genes.

### 2.4. Transcriptional Networks Governing Seed Coat Development: Roles of MYB, NAC, and WRKY Transcription Factors

Above the enzymatic level, seed-coat impermeability is likely orchestrated by a layered transcriptional network integrating secondary cell wall formation, phenylpropanoid metabolism, lipid polyester biosynthesis, and phenolic transport. In *Astragalus*, no MYB, NAC, or WRKY regulator has yet been functionally established as a direct controller of seed-coat-imposed dormancy [4]. Nevertheless, the regulatory logic emerging from other systems provides a strong conceptual framework. Among MYB factors, *MYB107* is one of the best-characterized regulators of seed-coat suberization. In *Arabidopsis*, *MYB107* positively regulates suberin biosynthetic gene expression during seed development, and disruption of *MYB107* increases permeability [25]. Closely related work further showed that *MYB107* and *MYB9* homologs jointly regulate suberin deposition in angiosperms, placing this MYB module near the core of protective barrier formation [26]. A recent PNAS study extended this concept by showing that cold-dependent seed dormancy can be promoted through polar lignification of a seed-coat suberin layer and that this barrier depends on *MYB107* [27].

NAC transcription factors are equally important because they often act as upstream master regulators of secondary cell wall biosynthesis. In seed-coat biology, this is well illustrated by *NST1*-like regulators. In hull-less pumpkin, a natural mutation in *NST1* arrested secondary cell wall formation in the seed coat and abolished proper lignin and cellulose deposition, demonstrating how a single NAC regulator can reorganize the entire structural program of testa development [28]. Although this work was not performed in legumes, the mechanistic implication is highly relevant: NAC regulators probably occupy an upper regulatory tier that determines whether palisade-like seed-coat cells complete their wall-thickening and barrier-formation program. In *Astragalus*, NAC-family orthologs should therefore be considered strong candidates for controlling both the anatomical differentiation and the biochemical hardening of the seed coat.

WRKY factors add another layer by linking seed-coat development with phenolic metabolism and specialized transport processes. In *Arabidopsis*, *Transparent testa glabra2* (*TTG2*) encodes a WRKY transcription factor required for normal seed-coat tannin and mucilage development [29]. Later work showed that *TTG2* regulates vacuolar transport steps in the proanthocyanidin pathway, including control over *TT12* and *AHA10*, thereby influencing how phenolic compounds are accumulated and compartmentalized during seed-coat maturation [30]. Although proanthocyanidin regulation is not identical to lignin or suberin biosynthesis, it is highly relevant to hardseededness because phenolics contribute to testa chemistry, wall sealing, and mechanical resistance. In *Astragalus*, WRKY-like regulators may therefore modulate the phenolic dimension of PY, especially where flavonoid deposition intersects with structural reinforcement and environmental resilience.

Overall, the most plausible model for *Astragalus* seed coat-imposed dormancy is a transcriptionally coordinated barrier program in which MYB regulators promote suberin and related aliphatics, NAC regulators drive wall differentiation and lignification, and WRKY regulators shape phenolic deposition and seed-coat maturation [27,28,30]. The final hardseed phenotype likely emerges only when these regulatory layers converge on the palisade layer and associated water-gap tissues. This layered model, in which phenylpropanoid, lignin, lipid-barrier, and transcription-factor modules converge on seed coat impermeability, is summarized in Figure 1A. What remains missing is direct *Astragalus* evidence at tissue resolution. Laser-capture transcriptomics, single-seed coat RNA-seq, spatial metabolomics, and comparative genomics across hard- and soft-seeded taxa should now make it possible to identify the lineage-specific regulators that convert a developing *Astragalus* testa into a fully water-impermeable structure.

## 3. Phytohormone Crosstalk and Signaling Networks (Regulating PD and Germination)

**Figure 2:**
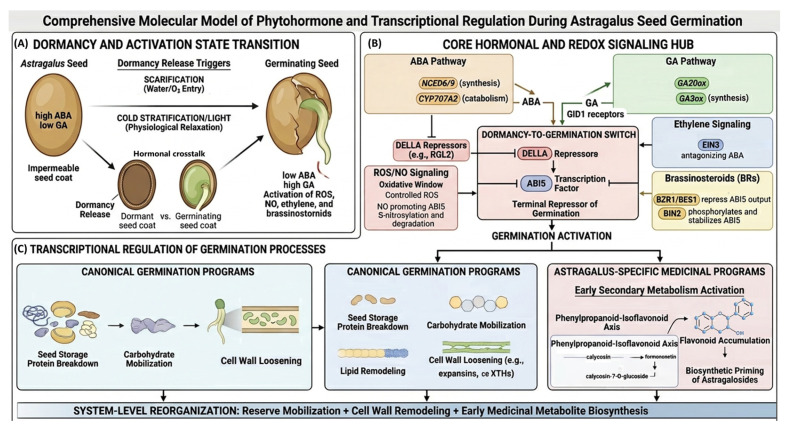
Comprehensive molecular model of phytohormonal and transcriptional regulation during *Astragalus* seed germination. The model integrates three interconnected modules. (**A**) Dormancy-to-activation transition. Dormant seeds are characterized by high ABA levels and an impermeable seed coat. Scarification enables water/O_2_ entry, reducing ABA and promoting the low-ABA/high-GA state that initiates germination. (**B**) Core hormonal and redox signaling hub. ABA and GA pathways converge on DELLA repressors (e.g., RGL2). ROS/NO signaling controls ABA catabolism (*CYP707A2*) and DELLA degradation via *S-nitrosylation*. GA signaling via *GID1* and BR signaling (*BZR1/BES1*) antagonize ABA output, governing the dormancy-to-germination switch. (**C**) Downstream transcriptional, metabolic, and medicinal-developmental outputs. Canonical programs induce storage protein breakdown, carbohydrate mobilization, and cell wall loosening. *Astragalus*-specific medicinal programs activate early secondary metabolism, including the phenylpropanoid/isoflavonoid axis, flavonoid accumulation, and biosynthetic priming of astragalosides.

In *Astragalus*, this section must be interpreted in the context of dormancy class. Because most species in the genus express physical dormancy (PY), hormonal and redox signaling become especially relevant after the water-impermeable seed coat has been breached, or in those taxa in which a physiological component persists in addition to PY [5]. This distinction is important. A meta-analysis across the genus showed that seed-coat-disruptive treatments such as mechanical or sulfuric-acid scarification are generally much more effective than prechilling or gibberellin application, indicating that in most *Astragalus* species hormonal stimulation alone cannot substitute for removal of the physical barrier [5]. Nevertheless, once imbibition proceeds, transcriptomic analysis in *A. mongholicus* reveals strong enrichment of plant hormone signal transduction and ABA-responsive pathways, showing that post-barrier germination in *Astragalus* is indeed governed by canonical signaling programs [31]. While seed-stage transcriptomic data are currently most comprehensive for *A. mongholicus*, supporting evidence from other *Astragalus* species is available through alternative approaches. Physiological studies in *A. membranaceus* have demonstrated that exogenous ABA significantly inhibits germination and alters reserve mobilization, confirming ABA’s functional role at the whole-seed level [32]. Additionally, transcriptomic analyses of bud dormancy transitions in *A. membranaceus* var. *mongholicus* have revealed similar hormone signaling pathway enrichment, suggesting that ABA/GA regulatory networks may be conserved across developmental contexts within the genus [12]. Collectively, these observations support the inference that once the physical seed-coat barrier is removed, ABA-GA antagonism functions as a central regulatory module in *Astragalus* germination, although further species-level transcriptomic studies would strengthen this generalization. The post-permeability signaling framework, including ABA-GA antagonism, redox regulation, and downstream germination outputs, is shown in Figure 2.

### 3.1. The Core ABA/GA Antagonism

Among endogenous regulators of seed dormancy and germination, the antagonism between abscisic acid (*ABA*) and gibberellins (*GA*) remains the central decision-making module [33,34,35,36]. In *Astragalus*, the available direct evidence aligns with this general paradigm. In *A. membranaceus*, exogenous ABA significantly inhibits both germination and post-germination growth, reduces seed-reserve mobilization efficiency, retards protein and lipid mobilization, and alters fatty-acid composition, demonstrating that ABA is not simply a passive dormancy correlate but an active suppressor of metabolic transition toward seedling establishment [32]. Likewise, germinating *A. mongholicus* seeds show enrichment of hormone-signaling and ABA-responsive modules during the early imbibition-to-germination sequence [31]. These observations strongly support the idea that once the testa constraint is removed, the ABA-to-GA balance becomes a major determinant of whether *Astragalus* seeds remain quiescent or progress to radicle protrusion [31,32].

At the metabolic level, dormancy release is typically associated with a shift from ABA accumulation toward ABA catabolism and GA biosynthesis. In *Arabidopsis*, NCED genes encode key rate-limiting enzymes of ABA biosynthesis, whereas *CYP707A* genes encode ABA 8′-hydroxylases that drive ABA catabolism [4]. Among them, *CYP707A2* plays a major role in the rapid decline in ABA during early imbibition, whereas *NCED6/NCED9* are major contributors to dormancy-associated ABA production [35]. Conversely, the activation of *GA20ox* and *GA3ox* underpins the production of bioactive GA, and this biosynthetic arm is a well-established counterpart to ABA decline during dormancy alleviation and germination [5]. Thus, the most plausible model for *Astragalus* is that, after permeability is acquired, successful germination requires both ABA depletion and GA biosynthetic activation, rather than a simple increase in GA alone [33,35]. Direct evidence for ABA’s inhibitory role during *Astragalus* germination is available from physiological studies in *A. membranaceus*, where exogenous ABA significantly inhibited germination and reduced reserve mobilization efficiency [36]. However, direct evidence for GA biosynthetic activation during *Astragalus* seed germination remains limited; the genus-wide meta-analysis by Soltani et al. (2020) found that gibberellin application was generally ineffective in breaking dormancy in *Astragalus* seeds [5], consistent with the predominantly physical nature of dormancy in the genus [2†L9–L16]. The transcriptomic evidence from *A. mongholicus* showing enrichment of hormone signaling pathways during germination [31] provides indirect support for the involvement of GA-related processes, but does not specifically demonstrate GA biosynthesis activation. We therefore present the ABA/GA antagonism model as a mechanistically grounded hypothesis for *Astragalus* that is supported by evidence from model systems and by partial evidence from the genus, but that awaits direct functional validation in *Astragalus* seeds.

The antagonism between ABA and GA is further sharpened at the signaling level through the interplay of DELLA repressors and ABI transcription factors, especially *ABI5* [34,36]. Under low-GA or high-ABA conditions, DELLA proteins such as *RGL2* remain stabilized and reinforce dormancy by stimulating ABA synthesis and promoting *ABI5* activity. ABI5 then functions as a terminal repressor of germination-related growth and metabolic programs [34,36]. When GA levels rise, GA perception by *GID1* receptors promotes DELLA degradation, releasing germination-associated growth potential and weakening the ABA-dominant state [33,36]. The ABA/GA node is therefore not just a hormonal ratio; it is a transcriptional and proteostatic switch in which DELLA stability and *ABI5* abundance jointly determine whether radicle emergence is repressed or permitted [33,36]. This central ABA-GA-DELLA-*ABI5* switch is depicted in Figure 2A,B.

For *Astragalus*, one practical implication is especially important. Across intact hard seeds, GA treatment is often ineffective or clearly secondary compared with scarification [5]. This means that in most species the ABA/GA antagonism should not be treated as the primary cause of non-germination in the dry, impermeable state. Rather, it becomes decisive after the physical barrier has been overcome, or in those seeds with a residual physiological block [5,31]. This nuance is essential for the genus: hormone signaling is central to germination control, but in many *Astragalus* taxa it operates downstream of coat-imposed dormancy release, not in place of it [5,31].

### 3.2. Synergistic Roles of Ethylene and Brassinosteroids in Promoting Radicle Protrusion

Ethylene is widely recognized as a positive regulator of dormancy release and germination in many species, largely because it antagonizes ABA action and interacts with both GA and ROS signaling [37]. Review-level evidence from *Arabidopsis* and other systems shows that ethylene-insensitive mutants are often more dormant or more ABA-sensitive, and that ethylene responsiveness depends strongly on dormancy status and seed physiological condition [37]. Mechanistically, the core ethylene pathway converges on *EIN2* and *EIN3*, which help translate gaseous ethylene signals into transcriptional programs favoring embryo growth and dormancy release. Although this pathway has not yet been dissected in *Astragalus* seeds, it is highly likely to function after imbibition in any *Astragalus* taxon where a physiological restraint remains after coat opening [37].

Brassinosteroids (BRs) provide a second pro-germination input. Classical work in *Arabidopsis* showed that BR can promote germination and partially rescue GA-deficient or GA-signaling-impaired backgrounds, indicating that BR contributes genuine germination-promoting activity rather than merely amplifying GA output [38]. In tobacco, BR and GA were shown to promote germination by distinct pathways, with BR acting more directly on embryo growth and endosperm rupture rather than fully substituting for the light- and GA-linked dormancy-release route [39]. This is particularly relevant to radicle protrusion, because the final transition to visible germination depends not only on hormonal status but on the mechanical ability of the embryo axis to overcome surrounding tissues. In that sense, BR can be viewed as a growth-permissive signal that helps convert hormonal derepression into actual emergence [38,39].

Importantly, the intersection of BR and ABA signaling involves *ABI5* as a central convergence point. In *Arabidopsis*, the negative BR signaling regulator *BIN2* directly phosphorylates and stabilizes *ABI5*, thereby reinforcing ABA-mediated inhibition of germination [40]. Conversely, BR-favored transcriptional regulators such as *BZR1* and *BES1* suppress ABA responses in part by repressing *ABI5*-linked transcriptional output [41]. These mechanistic studies in the model system establish a robust BR-*ABI5*-*BIN2* regulatory axis. For *Astragalus*, direct seed-specific molecular validation of this axis does not yet exist. However, transcriptomic observations during *A. mongholicus* seed germination indicate that post-imbibition phases can involve variable germination rates and differential stress sensitivity [31]. These observations raise the plausible hypothesis that a similar BR-*ABI5*-*BIN2* module may contribute to variation in germination vigor once the physical seed-coat barrier is relieved [31,36]. This candidate network, together with ethylene-mediated inputs, is integrated into the broader hormone-signaling hub shown in Figure 2B, where it converges with ABA-GA regulation to modulate germination competence.

### 3.3. Reactive Oxygen Species and Nitric Oxide as Crucial Signaling Hubs: Toward an “Oxidative Window” Model in Astragalus

Reactive oxygen species (ROS), especially hydrogen peroxide (H_2_O_2_), are now understood as dual-function molecules in seed biology: in excess they are damaging, but at controlled levels they act as central developmental signals [42,43]. Bailly and colleagues formalized this principle as the “oxidative window for germination,” a model proposing that germination proceeds only when ROS levels remain within a critical range bounded by lower and upper thresholds [42]. Later syntheses further emphasized that H_2_O_2_ participates in extensive crosstalk with ABA, GA, ethylene, and NO and must be tightly controlled in space and time to promote rather than inhibit germination [43]. Conceptually, ROS can therefore be viewed as a biochemical checkpoint that links embryo reactivation, cell wall loosening, redox balance, and hormonal signaling [42,43].

Nitric oxide (NO) is another major signaling hub in dormancy alleviation and germination. NO has been repeatedly identified as a potent dormancy-releasing factor in seeds, and one of its major pro-germination mechanisms is the weakening of ABA-dominant repression [44]. In *Arabidopsis*, NO promotes germination in part by triggering S-nitrosylation of *ABI5*, which facilitates *ABI5* degradation and thereby dismantles a key ABA-mediated brake on radicle protrusion [45]. NO also interacts positively with ethylene signaling. Under salt stress, its promotion of seed germination and seedling growth depends at least in part on *EIN3*, linking redox signaling to the ethylene transcriptional pathway [46]. Together, these findings show that ROS and NO are not peripheral stress by-products; they are integrative signaling nodes that can reshape hormonal state, transcriptional repression, and the mechanics of germination [43,46].

For *Astragalus*, the “oxidative window” should currently be treated as a mechanistic hypothesis with genus-level plausibility, not as a fully demonstrated seed-specific model. Direct molecular evidence for ROS- or NO-centered control in *Astragalus* seeds remains limited. However, the hypothesis is strengthened by two observations: first, studies cited in *A. mongholicus* germination work report that hydrogen peroxide can improve germination of hard *Astragalus* seeds under some conditions; second, once germination begins, *Astragalus* seeds clearly engage hormone-signaling and ABA-responsive programs [31]. A reasonable working model is therefore that, after coat rupture or scarification-mediated water entry, *Astragalus* seeds pass through a redox-sensitive checkpoint in which controlled ROS accumulation and NO production help tip the balance away from *ABA/ABI5* dominance and toward GA-supported embryo growth and radicle emergence. This proposed redox-sensitive checkpoint is summarized in Figure 2B, while its downstream effects on reserve mobilization, cell wall loosening, and medicinal-metabolite activation are shown in Figure 2C. Testing this model directly, through time-resolved redox imaging, hormone quantification, and transcriptomics of scarified versus intact seeds, would be a major step forward for the genus [31,42,46].

## 4. Molecular Responses to Dormancy-Breaking Treatments

Dormancy-breaking treatments in *Astragalus* should be interpreted through the lens of dormancy class. Because most species in the genus exhibit physical dormancy (PY), treatments that disrupt seed-coat impermeability usually have the strongest effect on germination, whereas temperature- and hormone-dependent responses become especially important in taxa that retain a physiological component after the physical barrier is breached [5,8]. A meta-analysis across the genus showed that mechanical and chemical scarification are generally among the most effective interventions, while the need for cold stratification is more restricted and appears tied to species with combinational dormancy rather than to *Astragalus* as a whole [8]. This distinction is crucial for molecular interpretation: different treatments do not merely “improve germination,” but activate different entry points into the dormancy-to-germination program. These treatment-dependent entry points into germination are positioned within the dormancy-release sequence in Figure 1B.

### 4.1. Scarification (Mechanical/Chemical): Wound Signaling, Water Channel Activation, and Rapid Hydration Responses

In *Astragalus*, scarification acts first by removing the physical blockade to water uptake. The clearest direct evidence comes from *A. adsurgens*, in which impermeable seeds became permeable after acid treatment, and scanning electron microscopy showed that the opening created by treatment was associated mainly with the hilum and extra-hilar region, through which water then entered the seed [13]. This demonstrates that scarification is not simply a nonspecific injury, but a targeted structural conversion of the seed coat from a sealed to a hydraulically active state. Once the testa is breached, the seed rapidly transitions from quiescence to imbibition-driven metabolism, oxygen entry, and embryo reactivation. This scarification-driven shift from physical exclusion to hydration competence is represented in Figure 1B.

At the molecular level, direct *Astragalus* evidence for post-scarification signaling remains limited, but the most plausible immediate downstream response is a combination of rapid rehydration, redox activation, and local damage-associated signaling. In plants more broadly, mechanical injury is known to trigger early signaling events involving reactive oxygen species (ROS), ion fluxes, jasmonate-related responses, ethylene-associated signaling, and electrical or membrane-based cues [47,48]. In the seed context, early imbibition is itself accompanied by ROS-linked signaling and metabolic activation rather than being a purely passive physical process [48]. Thus, in *Astragalus*, scarification should be viewed as a dual trigger: it physically permits water entry and likely also initiates a localized wound-like signaling environment that helps shift the seed from dormancy maintenance to germination competence.

Aquaporins are likely to be important in this transition, even though they have not yet been directly characterized in scarified *Astragalus* seeds. Seed-focused reviews show that aquaporins are deeply involved in germination-associated water movement, and later work demonstrated that aquaporins can influence seed dormancy and germination under stress conditions [49]. In practical terms, once scarification opens a water-entry site, plasma membrane and vacuolar aquaporins are likely to facilitate cell-to-cell redistribution of water, vacuolation, and embryo-axis expansion. This makes aquaporin activation a strong mechanistic candidate for the rapid hydration response that follows scarification in *Astragalus*, even though direct seed-stage validation remains to be performed in the genus.

### 4.2. Temperature Perception: Cold Stratification-Induced Transcriptional Reprogramming and Release of Physiological Dormancy

Cold stratification is not a universal requirement in *Astragalus*, but it becomes highly relevant in species with a physiological dormancy component superimposed on PY. The strongest example is *A. filipes*, where germination studies support combinational dormancy, and previous positive responses to cold stratification are specifically noted as evidence for that physiological component [8]. The genus-wide meta-analysis also points to cases in which scarification alone is insufficient and a further period of cold exposure appears necessary for full dormancy release [5]. By contrast, in strongly hard-seeded species such as *A. arpilobus*, neither high nor low temperature exposure was effective in breaking dormancy before scarification, underscoring that temperature cannot substitute for coat disruption where PY is dominant [7].

Where cold stratification is effective, it is best interpreted as a trigger of transcriptional and hormonal reprogramming rather than as a purely physical treatment. In seed biology more broadly, stratification releases dormancy through a coordinated shift in the ABA/GA system, including a decline in ABA dominance, enhancement of GA responsiveness, and changes in expression of genes linked to hormone metabolism and signaling [50,51]. In *Arabidopsis*, cold imbibition increases the accumulation of *GID1* receptor transcripts, and stratification together with after-ripening enhances GA sensitivity, highlighting that dormancy release depends not only on hormone amounts but also on signaling competence [51]. Accordingly, in *Astragalus* species with residual PD, cold stratification is most plausibly acting through a conserved reprogramming module that reduces ABA restraint and increases GA responsiveness after permeability is acquired.

Direct transcriptome-scale evidence for cold stratification in *Astragalus* seeds is still scarce, but available seed-germination transcriptomics in *A. mongholicus* show that plant hormone signal transduction is strongly engaged during the germination sequence [31]. This supports the broader inference that once the physiological block begins to relax, *Astragalus* seeds enter the same conserved regulatory space described in model systems, centered on hormone signaling, reserve mobilization, and embryo growth. For the genus, an important next step will be to compare scarified-only seeds with scarified-plus-stratified seeds in combinationally dormant taxa, so that the transcriptional signature of the physiological component can be separated from the purely structural release of PY.

### 4.3. Light Signaling: Phytochromes and PIFs Modulating Germination in Light-Responsive Astragalus Species

Light responses in *Astragalus* appear to be species-specific rather than uniform across the genus. In *A. membranaceus*, seeds germinated better under light than in darkness, and low temperature combined with light supported optimum germination in the tested conditions [52]. In contrast, scarified seeds of *A. arpilobus* germinated to high percentages in both light and darkness, indicating little or no obligate light requirement once physical dormancy had been removed [7]. Moreover, the recent *A. mongholicus* germination study notes previous evidence that full light and complete darkness had no significant effect on germination in that system [31]. Together, these data indicate that light sensitivity exists in some *Astragalus* species, but cannot yet be generalized as a shared genus-wide germination rule.

Where light does matter, the most likely regulatory architecture in *Astragalus* is the conserved phytochrome–PIF–ABA/GA module established in model plants. In *Arabidopsis*, phytochrome B (phyB) is a major promoter of light-induced germination, acting largely by destabilizing *PIF1*, a key repressor that favors the dormant state [36,53]. PIF1 represses germination by promoting ABA-associated outputs and constraining GA-promotive pathways, while light-activated phytochromes relieve this repression by triggering PIF degradation [36,53]. Additional work showed that *ERF55* and *ERF58* also repress light-induced germination partly through this same network [54]. This provides a compelling conceptual framework for light-responsive *Astragalus* species: where positive photoblasty is observed, it likely reflects a shift from PIF/*ABI5*-dominated repression in darkness toward phytochrome-mediated derepression under permissive light.

A key point for *Astragalus* is that light signaling probably acts after or alongside dormancy release, not in place of it. In strongly physically dormant seeds, light cannot act on an embryo that has not yet imbibed. Thus, phytochrome signaling becomes most relevant either in already permeable seeds, in scarified seeds, or in taxa whose dormancy includes a physiological light-sensitive component. This helps explain why some species show strong scarification responses with little light dependence, whereas others display additional light modulation once the coat barrier is no longer limiting. For future work, resolving this question will require species-specific experiments that combine permeability state, light regime, and transcript profiling of PHY, PIF, *ABI5*, and GA/ABA marker genes during the earliest hours after imbibition.

## 5. Omics-Based Insights into the Dormancy-to-Germination Transition

The dormancy-to-germination transition in *Astragalus* is increasingly accessible to transcriptomic and metabolomic profiling. At present, the strongest direct seed-stage resource is the recent time-course transcriptome of *Astragalus mongholicus* seeds [31], whereas broader genome-enabled pathway dissection has largely been developed from medicinal tissues such as roots, fruits, and plantlets rather than seeds themselves [55,56,57]. This creates an important interpretive boundary for the current study. Germination-related energy metabolism and reserve mobilization are supported by direct seed transcriptomic evidence, whereas the early activation of specialized metabolite biosynthesis, especially astragalosides, is still best treated as a mechanistically grounded but only partially validated inference. We acknowledge that true multi-omics integration, combining transcriptomics, proteomics, and metabolomics within a single experimental design, has not yet been achieved for *Astragalus* seeds. The present chapter therefore focuses primarily on transcriptomic evidence, complemented by available metabolomics data where applicable. The major molecular layers discussed, including transcriptional activation, reserve mobilization, and medicinal-metabolite biosynthesis, are summarized in Figure 1C.

### 5.1. Transcriptomics: Time-Course mRNA Profiling, Key DEGs, and the Emergence of Growth Programs

The most important direct transcriptomic study of *Astragalus* seed germination is the 0, 12, 24, and 48 h RNA-seq time course in *A. mongholicus* by Li et al. [31]. That study showed that the major transcriptional changes during germination are enriched in plant hormone signal transduction, plant-pathogen interaction, and metabolic activation, with GO terms linked to the generation of precursor metabolites and energy prominently represented [31]. Network analysis further identified a small set of candidate hub genes, including Cluster-28,554.0, *FAS4*, T10O24.10, and *EPSIN2*, indicating that *Astragalus* germination involves coordinated regulation of signaling, membrane dynamics, and energetic reactivation rather than merely passive imbibition [31]. In the context of seed-omics trends more broadly, this fits well with recent syntheses showing that seed germination is increasingly understood through multi-layered molecular profiling rather than single-pathway analysis [31,55].

What the current *Astragalus* seed transcriptome still does not resolve well is the cell-wall remodeling program that enables radicle protrusion. Here, recent comparative seed omics from non-*Astragalus* systems provide an important framework. Spatial transcriptomics in germinating barley showed that genes involved in cell wall metabolism are activated early, and that *alpha expansin 2* becomes strongly expressed around the radicle expansion zone during late germination, directly linking wall loosening to embryo emergence [56]. Likewise, combined transcriptome-proteome analysis in warm-stratified *Amomum tsaoko* identified XTH/EXP-related proteins among dormancy-release-associated regulators, together with hormone, MAPK, storage-reserve, and energy-metabolism pathways [58]. Thus, although expansins and xyloglucan endotransglycosylase/hydrolases (XTHs) have not yet been directly validated in *Astragalus* seeds, they should be regarded as high-confidence candidate modules for the biomechanical phase of germination.

### 5.2. Proteomics: Mobilization of Seed Storage Proteins and Post-Translational Control

Compared with transcriptomics, proteomic data for *Astragalus* seeds remain limited. However, physiological work on *A. membranaceus* already outlines the core metabolic logic expected from seed proteomics. During germination, starch and protein are mobilized rapidly, whereas lipids are used predominantly during post-germination growth; moreover, exogenous ABA and MeJA reduce reserve mobilization efficiency and retard protein and lipid utilization [32]. Although this study did not use large-scale proteomics, it strongly implies that germination in *Astragalus* depends on a coordinated program of storage-protein breakdown, carbohydrate release, lipid remodeling, and enzyme activation [32]. These results are highly informative from a proteomic perspective because they imply that early *Astragalus* germination depends on a tightly regulated sequence of storage-protein breakdown, carbohydrate release, lipid remodeling, and enzyme activation, even though the proteins themselves have not yet been globally profiled in the species.

While direct seed proteomic data for *Astragalus* remain unavailable, the expected functional logic can be inferred from well-characterized model systems. In *Arabidopsis*, proteome-scale analysis established that germination depends heavily on selective mRNA translation and protein turnover, rather than on transcript abundance alone [59]. More recent reviews of seed post-translational regulation emphasize that phosphorylation, ubiquitination, and related PTMs are central to germination control, especially through regulators such as ABI5- and DELLA-associated pathways [60]. These findings from model systems provide a strong hypothesis-generating framework for *Astragalus*; the mobilization of storage reserves is likely coordinated with PTM-dependent activation of hydrolases, glycolytic enzymes, and redox enzymes. However, we emphasize that direct proteomic validation in *Astragalus* seeds remains a priority for future research. The studies cited here from *Arabidopsis* [59,60] are included as mechanistic precedents that inform experimental design, not as direct evidence for *Astragalus* seed biology. In practical terms, this means that future *Astragalus* seed proteomics should not only quantify the loss of seed storage proteins (SSPs), but also identify PTM-dependent activation of hydrolases, glycolytic enzymes, redox enzymes, and signaling proteins that convert released reserves into embryo growth [59,60].

Broader seed proteomics shows what the next layer in *Astragalus* is likely to look like. In wheat, dynamic proteome analysis during germination identified proteins involved in storage degradation, carbohydrate metabolism, stress/defense, transcription/translation, and morphogenesis, and also showed that several reserve-related proteins such as globulin 3, sucrose synthase, beta-amylase, and AGPase are phosphorylated during germination. In rice embryos, phosphoproteomics demonstrated that phosphorylation enhances the activity of several germination-relevant enzymes, including fructokinase, pyruvate kinase, malate dehydrogenase, ascorbate peroxidase, and glutathione S-transferase. *Arabidopsis* dynamic proteomics, in turn, emphasized that selective mRNA translation and protein turnover are central to germination. Taken together, these studies strongly suggest that in *Astragalus*, future seed proteomics should focus not only on the disappearance of seed storage proteins, but also on the PTM-dependent activation of hydrolases, glycolytic enzymes, redox enzymes, and signaling regulators that convert stored reserves into embryo growth.

### 5.3. Metabolomics: From Reserve Degradation to Early Medicinal-Metabolite Biosynthesis

One of the most biologically distinctive features of *Astragalus* germination is that the transition from dormancy to seedling establishment appears to involve not only canonical reserve mobilization, but also the early activation of medicinally important secondary-metabolite pathways. This feature potentially distinguishes medicinal *Astragalus* seeds from many conventional crop-seed systems, in which germination is typically discussed primarily in terms of carbohydrate utilization, lipid turnover, and embryo growth [55,61]. Current evidence is strongest for the early recruitment of the flavonoid/isoflavonoid biosynthetic network during germination and early post-germination development. In *Astragalus membranaceus* and *A. membranaceus* var. *mongholicus*, Yang et al. demonstrated that germination is accompanied by increased accumulation of major medicinal isoflavonoids, including formononetin, calycosin, and calycosin-7-O-glucoside, together with transcriptional changes in flavonoid-biosynthetic genes [62]. Importantly, the authors showed that distinct biosynthetic strategies may operate in different *Astragalus* genotypes: in var. *mongholicus*, germination-associated flavonoid accumulation was linked predominantly to upstream pathway activation, whereas in *A. membranaceus* broader pathway-wide transcriptional activation was observed [62]. These findings provide direct evidence that flavonoid metabolism is dynamically recruited during early stages of *Astragalus* germination rather than being restricted to later vegetative or root developmental phases. This demonstrated early activation of the flavonoid/isoflavonoid axis is highlighted as a medicinal-developmental feature in Figure 1D and Figure 2C.

Recent multi-omics studies further reinforce the centrality of the flavonoid/isoflavonoid axis in *Astragalus* biology. Integrated transcriptomic-metabolomic analyses identified multiple core phenylpropanoid and isoflavonoid biosynthetic genes, including *PAL*, *C4H*, *4CL*, *CHS*, *CHI*, *IFS*, *F3H*, and *FLS*, whose expression patterns strongly correlate with flavonoid accumulation dynamics [56]. Likewise, full-length transcriptomics in *A. mongholicus* resolved extensive transcriptional networks associated with isoflavonoid biosynthesis, including numerous MYB, bHLH, AP2/ERF, and bZIP transcription factors potentially involved in pathway regulation [57], while its reference-grade genome demonstrated expansion of gene families related to triterpenoid and flavonoid biosynthesis, including tandemly expanded phenylpropanoid-associated families [10]. More recently, transcriptome-guided functional analyses showed that red-light exposure promotes calycosin accumulation through strong induction of *AmI3’H*, while *AmbHLH30* emerged as a candidate upstream regulator of calycosin biosynthesis [63]. Together, these studies support a model in which *Astragalus* tissues possess a responsive phenylpropanoid-isoflavonoid regulatory system that can be activated during developmental transitions, including germination and early seedling establishment [10,56,57,63].

Importantly, comparative evidence from other legumes also supports the concept that germination-associated activation of isoflavonoid metabolism may represent a broader adaptive strategy in medicinal or stress-responsive Fabaceae species. Integrated transcriptomic and metabolomic analyses of germinating alfalfa (*Medicago sativa*) sprouts revealed significant enrichment of flavonoid and isoflavonoid biosynthesis pathways during early germination, with increased accumulation of compounds such as calycosin, daidzein, and genistein under light-responsive conditions [64,65]. These observations strengthen the biological plausibility that rapid activation of phenylpropanoid-derived metabolites during germination contributes not only to secondary metabolism, but also to antioxidant buffering, cellular protection, and developmental acclimation during the vulnerable seed-to-seedling transition. In contrast, the evidence supporting early astragaloside accumulation during seed germination remains substantially more limited, and this distinction should be recognized clearly. Although recent genomic and transcriptomic studies have greatly expanded understanding of the triterpenoid–saponin biosynthetic machinery in *Astragalus* [10,57], most available evidence derives from roots, mature medicinal tissues, hairy-root systems, or whole-plant developmental analyses rather than directly from germinating seeds. Genome-enabled pathway analyses in *A. mongholicus* identified extensive expansion of gene families associated with triterpenoid and flavonoid biosynthesis and demonstrated strong tissue specialization in the accumulation of medicinal metabolites [10]. Similarly, transcriptomic studies resolved multiple candidate genes involved in triterpenoid–saponin biosynthesis, including cytochrome P450s, glycosyltransferases, and transcription-factor networks potentially regulating astragaloside production [57]. However, direct metabolomic evidence demonstrating substantial astragaloside accumulation during the earliest stages of seed germination is still lacking.

So far, there is still no equally direct seed-stage metabolomic study demonstrating that astragalosides increase during the first hours or days of *Astragalus* germination in the same way that flavonoids do [10,62,63]. What recent studies do provide is a strong biosynthetic scaffold. Full-length transcriptomics identified multiple candidate genes and gene families associated with triterpenoid–saponin biosynthesis [57], while the reference genome linked the high accumulation of triterpenoids and flavonoids mainly to root tissues and to the expansion of relevant biosynthetic families [10]. Thus, the most evidence-based interpretation at present is that germination in *Astragalus* likely involves early biosynthetic priming of triterpenoid–saponin pathways rather than immediate large-scale astragaloside accumulation. Under this framework, germination-associated signaling and metabolic reactivation may establish the transcriptional and enzymatic competence required for subsequent astragaloside biosynthesis during root differentiation and early seedling establishment, whereas flavonoid/isoflavonoid activation appears to occur more directly and immediately after dormancy release [10,57]. Framing the current evidence in this manner preserves the novelty of the medicinal-metabolite perspective while maintaining an appropriate distinction between experimentally demonstrated mechanisms and biologically plausible but not yet fully validated hypotheses. Accordingly, Figure 2C distinguishes direct flavonoid/isoflavonoid activation from the more tentative priming of triterpenoid–saponin pathways.

### 5.4. Next-Generation Integrative Omics: Spatial, Single-Cell, and Compartment-Resolved Perspectives on the Dormancy-to-Germination Transition

Although recent transcriptomic studies have substantially improved our understanding of germination-associated molecular dynamics in *Astragalus*, current datasets remain largely based on bulk tissue analysis, in which signals from multiple seed compartments are averaged into a single molecular profile [31]. While such datasets are valuable for identifying global regulatory trends, they inherently obscure the spatial and cell-type-specific processes that govern dormancy release and germination progression. Modern seed biology increasingly recognizes that germination is not a uniform whole-seed event, but a highly compartmentalized developmental transition involving distinct molecular programs operating in the seed coat, endosperm, cotyledons, embryonic axis, radicle meristem, and emerging seedling tissues [66,67,68].

This limitation is particularly important for *Astragalus*, where dormancy is predominantly imposed by the seed coat barrier rather than by embryo quiescence alone. In such systems, the earliest biologically decisive events are likely to occur in structurally specialized testa regions, including the hilum, lens, micropyle-associated tissues, and extra-hilar permeability domains [69]. Bulk RNA-seq cannot resolve whether transcriptional activation begins in the seed coat itself, within embryo tissues responding to hydration, or in localized signaling centers associated with radicle emergence. Thus, while the recent *A. mongholicus* time-course transcriptome provides a valuable systems-level overview [31], it cannot yet define the precise spatial origin of germination-regulatory signals.

Recent advances in spatial transcriptomics provide a transformative opportunity to overcome this limitation. In barley, spatially resolved transcriptomics of germinating grains revealed highly localized activation of cell wall remodeling, metabolic reprogramming, and tissue-specific transcriptional networks, demonstrating that germination is organized through sharply compartmentalized molecular domains rather than uniform transcriptional activation [68,70]. Similarly, high-resolution spatial atlases in *Arabidopsis* and cereal seeds have shown that embryo expansion, reserve mobilization, and stress-response signaling emerge in discrete tissue territories with distinct temporal dynamics [66,68]. Applying similar approaches to *Astragalus* would allow direct identification of the seed compartments initiating dormancy release, particularly whether permeability acquisition triggers local molecular reprogramming in testa tissues or whether embryo-derived signaling dominates the transition.

An equally important frontier is single-cell and single-nucleus omics, which have fundamentally reshaped understanding of developmental transitions in plant biology. Single-cell transcriptomics enables the dissection of heterogeneous tissues into distinct cellular populations, allowing identification of rare signaling centers, transitional cell states, and regulatory trajectories that are invisible in bulk analysis [71]. In the context of seed germination, this approach could distinguish transcriptional programs operating in epidermal testa cells, palisade layers, cotyledon parenchyma, embryo axis cells, vascular initials, and radicle meristematic populations. For *Astragalus*, such resolution would be especially informative because physical dormancy raises a central unresolved question: which cell populations first perceive successful dormancy release and initiate the molecular cascade toward germination?

Beyond transcriptomics, tissue-resolved metabolomics represents another critical but currently missing dimension. Germination-associated metabolism is often treated as a whole-seed phenomenon, yet metabolite accumulation is highly spatially structured. In medicinal *Astragalus*, this issue becomes particularly important because the biological novelty of this genus lies partly in the activation of pharmacologically relevant secondary metabolism during early development. Current evidence supports early flavonoid/isoflavonoid induction during germination [32,72], but the tissue localization of these metabolites remains unknown. It is unclear whether these compounds accumulate preferentially in cotyledons, embryonic axes, protective peripheral tissues, or rapidly differentiating seedling structures. Techniques such as mass spectrometry imaging (MSI), MALDI-based metabolite mapping, and tissue-specific metabolomics could directly resolve where medicinal metabolite biosynthesis is initiated and how this intersects with developmental and stress-responsive functions [73,74].

A compartment-resolved framework is also essential for understanding reserve mobilization dynamics. Starch degradation, protein hydrolysis, lipid remodeling, ROS production, and hormonal signaling are unlikely to occur synchronously across all tissues. Instead, recent seed systems biology strongly suggests sequential activation of specialized compartments, with embryo growth zones, storage tissues, and protective interfaces displaying distinct metabolic programs [66,67]. In *Astragalus*, resolving this spatial logic would clarify whether medicinal metabolite activation represents a direct component of embryo developmental programming or a secondary response associated with tissue differentiation and oxidative buffering.

Ultimately, the future of *Astragalus* seed biology lies in moving from conventional bulk omics toward a multi-scale systems framework integrating bulk transcriptomics, spatial transcriptomics, single-cell transcriptomics, phosphoproteomics, tissue-resolved metabolomics, and microbiome-informed ecological profiling. Such studies would help identify which seed tissues initiate dormancy release, which cells respond first to hydration, and where medicinal metabolites begin to accumulate. This next-generation, compartment-resolved omics direction is incorporated into Figure 1C,D as a future framework for linking seed tissues, molecular regulation, and medicinal-metabolite outputs. For *Astragalus*, this is especially compelling because it would allow direct testing of one of the central hypotheses emerging from this review, that successful germination is not merely a transition from dormancy to growth, but a coordinated developmental reprogramming in which structural barrier release, compartment-specific signaling, reserve mobilization, and early medicinal-metabolite activation unfold across precisely organized spatial domains [67,68,69,73,74].

A recurring challenge in *Astragalus* seed biology is distinguishing between mechanisms that have been directly experimentally verified in the genus and those that are inferred from studies in model plants or related species. To address this, we provide Table 1, which categorizes the key genes, pathways, and regulatory mechanisms discussed throughout this review according to their current evidence status in *Astragalus*. This table is intended to clarify the evidentiary basis for each mechanism and to highlight priority areas for future functional validation.

## 6. Current Challenges and Future Perspectives

Despite the rapid accumulation of anatomical, physiological, transcriptomic, metabolomic, and now genomic information about *Astragalus*, a major translational gap remains between candidate discovery and causal validation. Recent studies have already generated a seed-germination time-course transcriptome for *A. mongholicus*, chromosome-scale and telomere-to-telomere genome assemblies for *A. membranaceus*, and integrated omics resources for metabolite biosynthesis and dormancy-related transitions [11,31,75]. Yet a recent review focused specifically on seed dormancy and germination in *A. membranaceus* and *A. mongholicus* concluded that important bottlenecks still include incomplete elucidation of molecular mechanisms and insufficient understanding of key interacting factors [12,75,76,77]. In practice, this means that the field is currently far better at identifying correlated genes, pathways, and metabolites than at proving which of them actually determine hardseededness, residual physiological dormancy, or uniform germination performance in vivo [11,31,75]. The transition from descriptive omics toward functional validation, epigenetic analysis, and breeding-oriented applications is summarized in Figure 1D.

### 6.1. Bottlenecks: Over-Reliance on Descriptive Omics Data and Limited Functional-Validation Platforms

The first major bottleneck is the continued over-reliance on descriptive omics. Time-course RNA-seq, weighted gene co-expression network analysis, genome assembly, and pathway annotation are now available for medicinal *Astragalus* taxa, but these datasets mainly provide candidate lists rather than mechanistic proof [11,31,75]. This is not a trivial limitation. Seed dormancy and germination are multilayered traits integrating seed coat anatomy, hormone balance, redox signaling, reserve mobilization, and environmental sensitivity. In such systems, co-expression alone cannot establish whether a gene is causal, compensatory, or simply downstream of germination onset. Thus, one of the most pressing needs in *Astragalus* biology is to move from “which genes change” to “which genes matter”.

A second bottleneck is that the published toolset for *Astragalus* still appears to be concentrated mainly in hairy-root systems, tissue culture, and metabolite-engineering platforms, rather than in routine whole-plant reverse genetics. The literature clearly documents successful *Agrobacterium rhizogenes*-mediated hairy root production in *A. membranaceus* and its use for pathway engineering and metabolite enhancement [78,79]. However, the broader genomic literature continues to emphasize that available genetic and genomic information has been limited and that new assemblies are expected to facilitate future functional studies, implying that the enabling infrastructure is still catching up with the omics data flood [11,75]. Put differently, *Astragalus* now has many of the maps, but still too few of the tools needed to test the routes. This mismatch is probably the single biggest reason why dormancy research in the genus remains largely correlative [11,75,78].

A related issue is the absence, in the literature I found, of the kind of community-scale mutant resources that have driven functional breakthroughs in classic model systems. Rather than claiming that such resources do not exist anywhere, the safer conclusion is that current published *Astragalus* resources are still centered on assemblies, transcriptomes, pathway analyses, and transformation-derived root systems, not on widely used mutant libraries for seed biology [11,31,78,79]. That makes forward and reverse genetic dissection slower, more fragmented, and more dependent on indirect inference than it should be.

### 6.2. Moving Toward Functional Genomics: CRISPR/Cas9 and Agrobacterium rhizogenes-Mediated Assays

The most realistic near-term path forward is not to wait for a perfect stable transformation pipeline, but to use tiered functional genomics. The first tier is already available: *Astragalus* hairy-root systems have been established and exploited for specialized-metabolite studies, including isoflavonoid and astragaloside work [78,79]. In 2024, an efficient hairy-root induction system in *A. membranaceus* was reported together with significant enhancement of astragalosides through overexpression of *AmUGT15*, directly demonstrating that transformed root platforms can be used not just for metabolite accumulation but for gene-function manipulation in this genus [78]. Although roots are not seeds, this matters for dormancy research because the same platform can be used to test regulators of hormone signaling, phenylpropanoid flux, lipid barrier formation, and downstream medicinal-metabolite pathways much faster than whole-plant transformation would allow [78,79].

The second tier is the integration of these root platforms with *CRISPR/Cas9*. In other plant systems, hairy-root transformation combined with *CRISPR/Cas9* has already been shown to provide a fast and efficient route for testing editing constructs and studying target-gene function, including later regeneration and heritability analysis in some cases [80]. This has direct methodological relevance to *Astragalus*. Even if stable seed-to-seed editing remains difficult in the short term, *A. rhizogenes*-mediated composite systems could still be used to rapidly interrogate candidate genes emerging from dormancy omics, especially those involved in seed coat polymer deposition, hormone signaling, ROS homeostasis, or secondary-metabolite priming. In other words, the goal should be to build a validation pipeline, not just a single transformation method: candidate nomination by omics, rapid screening in composite/hairy-root assays, and then selective investment in stable editing only for the most decisive loci [78,80].

For seed dormancy specifically, this strategy also encourages a broader experimental logic. Genes controlling physical dormancy may be difficult to assay solely in root systems, but many downstream regulators of phenylpropanoid biosynthesis, suberin/cutin metabolism, transcriptional control, and hormone signaling can still be functionally probed in tractable tissues before moving into seed-focused validation. Thus, *CRISPR/Cas9* in *Astragalus* should be seen not as an all-or-nothing proposition, but as a staged entry into causal biology.

### 6.3. The Unexplored Epigenetic Frontier: DNA Methylation, Histone Modifications, and Non-Coding RNAs

A particularly promising but still underdeveloped frontier in *Astragalus* seed biology is epigenetic regulation. Reviews of seed germination increasingly emphasize that DNA methylation, histone modification, chromatin remodeling, and small/non-coding RNAs all contribute to the transition from dormancy to germination [81]. More importantly for an ecologically oriented genus such as *Astragalus*, recent work in model systems shows that maternal plants can establish an epigenetic state in progeny seeds that affects dormancy. In *Arabidopsis*, for example, a *VEL3* histone deacetylase complex was shown to establish a maternal epigenetic state controlling progeny seed dormancy [82]. This finding is conceptually important because it connects environmental history, maternal effects, and seed behavior through a molecular mechanism rather than through a vague “memory” metaphor [82].

For *Astragalus*, the implications are substantial. Many species occupy environmentally variable, drought-prone, saline, cold, or otherwise stressful habitats where the timing of germination has obvious fitness consequences. It is therefore reasonable to hypothesize that at least part of the inter-annual and inter-population variability in dormancy expression reflects epigenetic ecological memory, whether mediated through maternal environment, seed maturation temperature, stress history, or inherited chromatin states. At present, however, this remains almost completely unexplored in the genus. No integrated framework yet links *Astragalus* hardseededness or residual physiological dormancy to methylome dynamics, histone marks, or small-RNA networks. That gap is striking given how central these mechanisms are becoming in the broader seed field [81,82].

A productive next step would be to integrate bisulfite sequencing, chromatin profiling, and sRNA sequencing into dormancy-release experiments across contrasting maternal environments. Such work could test whether seeds produced under drought, salinity, or cool maturation conditions enter germination with different epigenetic states and whether those states alter permeability thresholds, ABA sensitivity, or redox behavior. This would move *Astragalus* seed biology from static trait description toward a much richer model in which dormancy is both genetically encoded and environmentally written.

### 6.4. Agronomic Translation: Toward Marker-Assisted Selection for Low Hardseededness and Uniform Germination

The final challenge is translation. For medicinal production, ecological restoration, and forage development, the desired phenotype is often not maximum dormancy, but predictable emergence, reduced hardseededness, and uniform germination. Agronomically, this makes breeding highly relevant. Even outside medicinal *Astragalus*, authors discussing forage *Astragalus* have explicitly identified selection for more soft-seeded accessions as a priority [83]. At the same time, new genomic resources and candidate-gene studies are beginning to supply the raw material for marker development. In *A. cicer*, a 2025 transcriptomic study identified candidate genes associated with high seedling vigor and explicitly suggested that these genes could be used as molecular markers for genetic improvement [84]. Together with the recent high-quality genome assemblies for medicinal *Astragalus*, this points toward a realistic path for marker-assisted selection and, eventually, genomic selection [11,83,84].

For dormancy traits, the breeding logic should be two-tiered. First, germplasm screening should identify naturally less hard-seeded, more uniformly germinating accessions without sacrificing vigor, seed longevity, or medicinal value. Second, once candidate loci are validated, breeders can deploy markers linked to seed coat structure, permeability behavior, hormone sensitivity, or early seedling vigor to accelerate selection. For medicinal *Astragalus*, the ideal target is not simply “soft seed,” but controlled and agronomically manageable dormancy: enough resilience for storage and field reliability, but not enough to create erratic establishment. This balance will be especially important if breeding is to preserve the distinctive secondary-metabolite profile that gives *Astragalus* its medicinal identity [11,75,83].

Overall, the future of *Astragalus* seed research will depend on how quickly the field can connect four currently separate layers: structural dormancy biology, seed-state omics, functional genomics, and breeding translation. The genus now has enough genomic foundation to make that transition feasible. What it still needs is a decisive methodological shift from descriptive cataloging to experimentally validated, trait-oriented seed biology [11,75,83,84]. The applied endpoint of this shift, including marker-assisted selection for reduced hardseededness and uniform germination, is summarized in Figure 1D.

## 7. Conclusions

Seed dormancy and germination in *Astragalus* represent an integrated biological process shaped by seed coat structure, hormonal regulation, metabolic reprogramming, and ecological adaptation. Across the genus, the dominant pattern is physical dormancy imposed by a water-impermeable seed coat, making hardseededness the first and most decisive barrier to germination. This structural control explains why scarification remains the most effective dormancy-breaking treatment in many species and why purely hormonal or environmental interventions often show limited success unless a physiological component also exists.

At the same time, germination in *Astragalus* is far more than a simple physical release from dormancy. Once the seed coat barrier is overcome, the transition to active growth depends on a coordinated regulatory network involving ABA-GA antagonism, additional contributions from ethylene and brassinosteroids, and the signaling functions of reactive oxygen species and nitric oxide. These pathways govern reserve mobilization, embryo reactivation, cell expansion, and the final emergence of the radicle. Thus, the dormancy-to-germination transition in *Astragalus* should be viewed as a two-level process in which structural release and molecular activation are tightly connected.

A particularly important insight emerging from recent studies is that medicinal *Astragalus* seeds differ conceptually from many conventional crop seeds. Germination is not only associated with degradation of stored reserves and activation of basic energy metabolism, but also with the early recruitment of pathways involved in characteristic secondary metabolites, especially flavonoids and isoflavonoids. This feature gives *Astragalus* seed biology a distinctive medicinal-developmental dimension and provides a valuable framework for linking seed physiology with the pharmaceutical value of the genus.

Despite recent progress, major gaps remain. Current research is still dominated by descriptive anatomy, physiology, and omics datasets, whereas direct functional validation of candidate genes remains limited. The next phase of the field should therefore move beyond cataloging responsive genes and metabolites toward causal analysis through integrative multi-omics, spatially resolved seed studies, functional genomics, and eventually breeding-oriented molecular tools. In particular, the roles of epigenetic regulation, ecological memory, and seed-associated microbiomes remain largely unexplored and may open important new directions for understanding variation in dormancy and germination behavior.

Overall, *Astragalus* is a useful model for linking hardseededness, medicinal-metabolite development, and germplasm utilization in legumes. A deeper mechanistic understanding of how hard seeds are unlocked will not only improve germplasm use, cultivation, and breeding, but will also broaden our general understanding of how medicinal legumes coordinate survival, germination, and specialized metabolism at the earliest stage of plant life.

## Figures and Tables

**Table 1:** Evidence status of molecular mechanisms discussed in the context of *Astragalus* seed dormancy and germination.

Mechanism/Pathway	Key Genes/Regulators	Verified in *Astragalus*?	Evidence Source	Ref.
Seed Coat Structure				
Palisade layer as water barrier	Macrosclereid cells	Verified	Anatomical observation (SEM) in *A. adsurgens*	[13]
Dehydration-dependent sealing	Seed moisture content	Verified	Demonstrated in *A. adsurgens*	[13]
Hilum/extra-hilar water entry sites	-	Verified	SEM evidence in *A. adsurgens*	[13]
Phenylpropanoid/Lignin Biosynthesis				
PAL, C4H, 4CL in seed hardening	*PAL*, *C4H*, *4CL*	Nil	Inferred from soybean, Brassica	[16,17]
CAD in monolignol production	*CAD*	Nil	Inferred from soybean, Brassica	[16,17]
Phenylpropanoid pathway regulation	-	Nil	Hypothesis based on related legumes	[6,18]
Lipid Metabolism (Suberin/Cutin)				
*CYP86B1* in suberin polyester	*CYP86B1*	Nil	Verified in *Arabidopsis*	[23]
*GPAT5* in suberin formation	*GPAT5*	Nil	Verified in *Arabidopsis*	[22]
*KCS12* in VLCFA synthesis	*KCS12*	Nil	Verified in *Medicago truncatula*	[20]
*LTPG15* in monomer export	*LTPG15*	Nil	Verified in *Arabidopsis*	[24]
ANR in flavonoid-lipid crosstalk	*ANR*	Nil	Verified in *Medicago truncatula*	[21]
*KNOX4* in cuticle development	*KNOX4*	Nil	Verified in *Medicago truncatula*	[19]
Transcription Factors				
*MYB107* in suberin regulation	*MYB107*	Nil	Verified in *Arabidopsis*	[26]
*MYB9* in suberin deposition	*MYB9*	Nil	Verified in *Arabidopsis*	[26]
*NST1* in secondary cell wall	*NST1*	Nil	Verified in pumpkin (seed coat)	[28]
*TTG2* (WRKY) in tannin/mucilage	*TTG2/WRKY*	Nil	Verified in *Arabidopsis*	[29,30]
ABA/GA Signaling				
ABA inhibits germination	-	Verified	Physiological evidence in *A. membranaceus*	[36]
ABA alters reserve mobilization	-	Verified	Physiological evidence in *A. membranaceus*	[36]
ABA/GA antagonism	-	Verified	Transcriptomic evidence in *A. mongholicus*	[31]
*NCED6/9* in ABA biosynthesis	*NCED6*, *NCED9*	Nil	Inferred from *Arabidopsis*	[34]
*CYP707A2* in ABA catabolism	*CYP707A2*	Nil	Inferred from *Arabidopsis*	[4]
*GA20ox/GA3ox* in GA biosynthesis	*GA20ox*, *GA3ox*	Nil	*Inferred from Arabidopsis*	[5]
*DELLA-ABI5* signaling	*RGL2*, *ABI5*	Nil	Inferred from *Arabidopsis*	[33,35]
Ethylene and Brassinosteroids				
Ethylene promotes germination	*EIN2*, *EIN3*	Nil	Inferred from *Arabidopsis*	[37]
BR promotes germination	*BIN2*, *BZR1*, *BES1*	Nil	Inferred from *Arabidopsis*	[38,39,40,41]
BR-ABI5-BIN2 axis	*BIN2*, *ABI5*, *BZR1*	Nil	Inferred from *Arabidopsis* (candidate network for *Astragalus*)	[40,41]
ROS/NO Signaling				
ROS oxidative window	-	Nil	Inferred from *Arabidopsis*	[42,43]
NO promotes germination via *ABI5* S-nitrosylation	*ABI5*	Nil	Verified in *Arabidopsis*	[45]
NO-ethylene crosstalk via *EIN3*	*EIN3*	Nil	Verified in *Arabidopsis*	[46]
Transcriptomics				
Hormone signaling enrichment in germination	-	Verified	RNA-seq in *A. mongholicus* seeds	[31]
Candidate hub genes (*FAS4*, *EPSIN2*, etc.)	*FAS4*, *EPSIN2*	Verified	RNA-seq in *A. mongholicus* seeds	[31]
Metabolomics/Secondary Metabolism				
Flavonoid/isoflavonoid activation in germination	-	Verified	Metabolomics in *A. membranaceus*	[62]
PAL, C4H, 4CL, CHS, CHI, IFS in flavonoids	Multiple genes	Verified	Transcriptomic/metabolomic in *A. membranaceus*	[56,62]
AmI3’H in calycosin biosynthesis	*AmI3’H*	Verified	Functional characterization	[63]
*AmbHLH30* as flavonoid regulator	*AmbHLH30*	Verified	Functional characterization	[63]
Astragaloside biosynthesis priming	-	Nil	Inferred (genomic/transcriptomic evidence from roots, not seeds)	[10,57]
Proteomics/PTMs				
Seed storage protein mobilization	-	Nil	Inferred from *Arabidopsis*, rice, wheat	[59,60]
Phosphorylation in germination enzymes	-	Nil	Inferred from rice, *Arabidopsis*	[59,60]

## Data Availability

No new data were created or analyzed in this study. Data sharing is not applicable to this article.

## Abbreviations

Abbreviation	Full Form
4CL	4-Coumarate:CoA ligase
ABA	Abscisic acid
ABI5	ABA insensitive 5
AGPase	ADP-glucose pyrophosphorylase
AmbHLH30	*Astragalus membranaceus* basic helix-loop-helix 30
AmI3’H	*Astragalus membranaceus* Isoflavone 3’-hydroxylase
AmUGT15	*Astragalus membranaceus* UDP-glucosyltransferase 15
ANR	Anthocyanidin reductase
AP2/ERF	APETALA2/ethylene-responsive factor
BES1	BRI1-EMS-suppressor 1
bHLH	Basic helix-loop-helix
BIN2	Brassinosteroid Insensitive 2
BR	Brassinosteroids
bZIP	Basic leucine zipper
BZR1	Brassinazole-Resistant 1
C4H	Cinnamate 4-hydroxylase
CAD	Cinnamyl alcohol dehydrogenase
CCoAOMT	Caffeoyl-CoA O-methyltransferase
CHI	Chalcone isomerase
CHS	Chalcone synthase
CRISPR	Clustered Regularly Interspaced Short Palindromic Repeats
CYP	Cytochrome P450
CYP707A	Cytochrome P450 707A
CYP86B1	Cytochrome P450 86B1
DEGs	Differentially expressed genes
EIN2	Ethylene insensitive 2
EIN3	Ethylene insensitive 3
EPSIN2	EPSIN-like protein 2
ERF	Ethylene response factor
ERF55/58	Ethylene Response Factor 55/58
F3H	Flavanone 3-hydroxylase
FAS4	Fatty acid synthase 4
FLS	Flavonol synthase
GA	Gibberellins
GA20ox	GA 20-oxidase
GA3ox	GA 3-oxidase
GID1	GA INSENSITIVE DWARF1 (GA receptor)
GO	Gene Ontology
GPAT	Glycerol-3-phosphate acyltransferase
GPAT5	Glycerol-3-phosphate acyltransferase 5
HCT	Hydroxycinnamoyl-CoA:shikimate hydroxycinnamoyl transferase
HDA15	Histone deacetylase 15
IFS	Isoflavone synthase
KCS	β-Ketoacyl-CoA synthase
KCS12	β-Ketoacyl-CoA synthase 12
KNOX	KNOTTED1-like homeobox
KNOX4	KNOTTED1-like homeobox 4
LTP	Lipid transfer protein
LTPG15	Glycosylphosphatidylinositol-anchored lipid transfer protein 15
MALDI	Matrix-Assisted Laser Desorption/Ionization
MAPK	Mitogen-activated protein kinase
MeJA	Methyl jasmonate
MSI	Mass spectrometry imaging
MYB	Myeloblastosis (transcription factor family)
MYB107/9	Myeloblastosis 107/9
NAC	NAM, ATAF1/2, and CUC2
NCED	9-cis-epoxycarotenoid dioxygenase
NCED6/9	9-cis-epoxycarotenoid dioxygenase 6/9
NO	Nitric oxide
NST1	NAC secondary wall thickening promoting factor1
*OsPsbS1*	*Oryza sativa* Photosystem II subunit S1
PAL	Phenylalanine ammonia-lyase
PD	Physiological dormancy
PIF	Phytochrome-interacting factor
PIF1	Phytochrome-interacting factor 1
PTM	Post-translational modification
PY	Physical dormancy
RGL2	RGA-LIKE 2
RNA-seq	RNA sequencing
ROS	Reactive oxygen species
SEM	Scanning electron microscopy
sRNA	Small RNA
SSPs	Seed storage proteins
TT12	transparent testa 12
TTG2	transparent testa glabra2
VEL3	vernalization 5/vin3-like 3
VLCFA	Very-long-chain fatty acid
WRKY	Named after conserved WRKYGQK amino acid sequence
XTH	Xyloglucan endotransglycosylase/hydrolase
